# Editorial: Vascular aging through understanding of inherited basis of aortic disease

**DOI:** 10.3389/fgene.2026.1778745

**Published:** 2026-01-29

**Authors:** Maya S. Safarova, Katherine Athayde Teixeira de Carvalho, Cornelie Nienaber-Rousseau

**Affiliations:** 1 Division of Cardiovascular Medicine, Department of Internal Medicine, Medical College of Wisconsin, Milwaukee, WI, United States; 2 Instituto de Pesquisa Pele Pequeno Principe, Curitiba, Brazil; 3 Centre of Excellence for Nutrition (CEN), Faculty of Health Sciences, North-West University, Potchefstroom, South Africa; 4 Medical Research Council Unit for Hypertension and Cardiovascular Disease, North-West University, Potchefstroom Campus, Potchefstroom, South Africa

**Keywords:** aortopathy, arterial aging, epigenetics (DNA methylation), polygenic DNA methylation alterations (methylation scores), prevention, progression and risk prediction, rare monogenic genetic alterations, vascular health and disease

## Introduction

Age estimation based on only one of the aging imaging or blood markers often fails to reflect the complexity of aging and can yield suboptimal assessments of biological age. An individual’s genetic predisposition establishes a lifelong baseline risk trajectory that can be altered by clinical, environmental, lifestyle and socioeconomic factors, guiding pharmacotherapy and lifestyle modifications. Conventional cardiovascular risk assessment tools and calculators use chronologic (biologic) age as the main driver of the cardiovascular disease burden. In clinical practice this assessment is further utilized for shared decision-making regarding initiation or postponing pharmacological and non-pharmacological interventions. However, such uniformed approach to cardiovascular prevention using traditional risk factors results in underdiagnosis and undertreatment in the large population of younger individuals and overtreatment in the elderly population.

Aorta-specific DNA methylation profiling, post-translational histone modifications, and non-coding RNAs can be used as potential tools to gain new insights into the pathogenesis of aortopathies and risk stratification (Maredia et al., 2021; Björck et al., 2018). Further, aorta-specific modulation of chromatin with an efficient and specific delivery system, as well as Reprogramming of a pathologic epigenetic landscape can serve as a potential therapeutic target, when approaching arterial aging. It has been shown that the same area accumulated at a younger age, compared with older age, resulted in a greater risk increase, emphasizing the importance of optimal risk factor control starting early in life. Coronary artery calcium, carotid plaque, and arterial stiffness have been proposed to be used as estimates of an arterial age in adults. However, the predictive value of these risk modifiers localized to a single vascular bed lacks specificity when taken in isolation, calling for more precise measures of vascular aging, especially in younger individuals.

## Genetic and epigenetic regulation of aortic pathophysiology

The epidemiology of the aortic disease in the population is poorly studied. Different segments of the aorta arise from distinct embryonic sources, accounting for heterogeneity in segmental gene expression and cell-subtype features. There are different models of aortic disease as a result of the loss of contractile function of smooth muscle cells and a phenotypic switch towards similar cells of different origins such as mesenchymal cells or myofibroblasts. Emerging research underscores gene-gene and gene–environment interactions impacting the trajectory of vascular aging (Corella and Ordovas, 2014). The epigenetic regulations, or “changes in non-DNA sequences,” (Waddington, 2012; Cavalli and Heard, 2019) are reversible and dynamically control gene expression. The abnormal methylation status of candidate genes for coronary heart disease, heart failure, and hypertension among others can be used as a marker to assess cardiovascular disease progression (Shi et al., 2022). In recent years, increasing evidence has accumulated for histone acetylation and miRNA in vascular calcification progression. Targeting epigenetic key enzymes, especially the DNA methyltransferases, histone methyltransferases, histone acetylases, histone deacetylases and their regulated target genes, could represent an attractive new approach to aortic disease and arterial aging in general.

## Highlights from the Research Topic

This *Research Topic* presents a collection of articles in the Cardio-Genomics space with an emphasis on the genetic basis and architecture of syndromic and non-syndromic aortic disease. Liu et al. used differentially expressed chromatin regulators to construct a nomogram and ascertain aortic dissection. Odogwu et al. reviewed transcriptome studies in humans and model systems of the spectrum of congenital heart disease, identifying critical gaps and therapeutic frontiers. Qin et al. explored X-chromosome gene expression related to sex dimorphism in ischemic stroke, offering novel perspectives on how sex-linked transcriptional regulation influences vascular and neurological outcomes. Tsuburaya-Suzuki et al. described the long-term course of adult-onset refractory epilepsy in a patient with cardiofaciocutaneous syndrome due to a pathogenic *MAP2K1* variant, expanding our understanding of genotype–phenotype correlations in syndromic aortopathies with neurological manifestations. Zhang et al. presented trio-exome sequencing analyses identifying a nonsense variation of the *NKX2-5* cardiac transcription factor in a family with nonsyndromic congenital heart disease, reinforcing the role of rare pathogenic variants in cardiac and vascular malformations.

## Future perspectives

**FIGURE 1:**
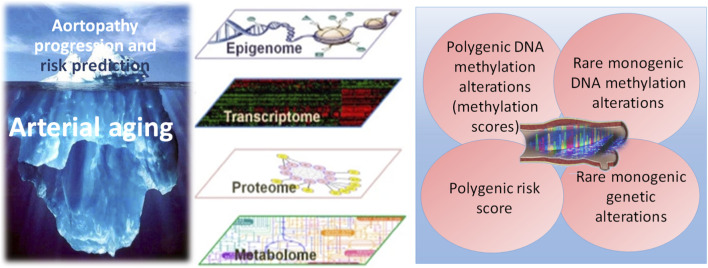
Personalised cardiovascular paradigm in aortic disease. An individual’s genetic predisposition establishes a lifelong baseline risk trajectory that can be altered by clinical, environmental, lifestyle and socioeconomic factors, potentially prompting and guiding pharmacotherapy and supporting lifestyle modifications. Epigenetics influences the aorta by regulating gene expression without changing the DNA sequence, playing a crucial role in aortic health and disease, such as aneurysms and dissections. This regulation is achieved through mechanisms such as DNA methylation, histone modifications, and non-coding RNAs. These processes are dynamic, making them a target for potential interventions promoting healthy aging.

Advances in high-throughput sequencing, chromatin profiling, and transcriptomics continue to uncover insights into vascular homeostasis across ancestral populations (Figure 1). Efforts should be made to expand representation across ethnicities, and provide functional validation of molecular findings in longitudinal cohorts. Precision cardio-genomics holds promise not only to improve risk prediction but also to pave the way for novel interventions that target arterial aging at its molecular roots.

## Conclusion

Bridging systems biology with clinical insights requires a multifaceted approach that involves data integration, computational modeling, and a cross-disciplinary dialogue to set the stage for a new era of a personalised cardiovascular paradigm. Ultimately, the approach in which biological, rather than chronological age is being used aims to transform the medicine by providing a mechanistic, network-based understanding of disease, allowing physicians to select optimal therapeutic regimens for individual patients.
